# Circumstances and causes of sudden circulatory arrests in the Dutch province of Limburg and the involvement of citizen rescuers

**DOI:** 10.1007/s12471-017-1057-1

**Published:** 2017-12-04

**Authors:** R. W. M. Pijls, P. J. Nelemans, B. M. Rahel, A. P. M. Gorgels

**Affiliations:** 1 Department of Cardiology, CAPHRI school for Public Health and Primary Care, Maastricht University Medical Centre+, Maastricht, The Netherlands; 2 Department of Epidemiology, CAPHRI school for Public Health and Primary Care, Maastricht University Medical Centre+, Maastricht, The Netherlands; 3 Department of Cardiology, Viecuri Medical Centre for Northern Limburg, Venlo, The Netherlands

**Keywords:** resuscitation, community responder, cause, circumstance

## Abstract

**Background:**

Recently we showed that a citizen volunteer system using text message alerts improves survival of out-of-hospital sudden circulatory arrest (OHCA). It is important to characterise the OHCA population encountered by the volunteers regarding circumstances and causes of the arrests.

**Methods and Results:**

Eligible for this study were 968 OHCAs that occurred between April 2012 and April 2014 in the Dutch province of Limburg. The distribution of causes of OHCA, patient characteristics and resuscitation settings were compared between 492 arrests wherein volunteers were notified and 476 arrests where the dispatcher decided not to do so.

In case of notification, the cause of OHCA was known in 345 cases and of cardiac origin (treatable) in 83.2% (287/345). About 41% of the cardiac arrests were caused by acute or chronic coronary artery disease. OHCA occurred within the home environment in about 84%. The OHCA was witnessed in 75% of the cases. In 60.9% of the cases a witness or bystander had already started basic life support. However, in approximately 18% of the OHCAs the volunteer was the first to start basic life support before arrival of the ambulance. In about 75% of the OHCAs the ambulance arrived at 6 minutes or later after time of notification by the dispatch centre.

**Conclusion:**

The volunteer system is predominantly activated in situations for which it was developed; cases with cardiac aetiology (58%) and cases in the home environment (84%). The majority of patients encountered by the volunteers had ‘hearts too good to die’, underscoring the benefit of deploying citizen rescuers in programs to improve survival of OHCA.

## Introduction

Recently we described that a novel citizen volunteers alert system significantly contributes to survival of out-of-hospital circulatory arrest (OHCA) of cardiac origin [1]. The contribution of the alert system to survival is most substantial in witnessed arrest, within the home environment, at slightly delayed arrival of the first ambulance and during the evening/night [2]. The zip-code based system was developed especially for OHCA within the home environment, enabling the dispatch centre to alert trained citizen rescuers simultaneously with the ambulances. Involving citizens as first responders in resuscitation of cardiac arrest, imposes them with a large responsibility. It is therefore crucial to study whether they indeed encounter emergency cases with a reasonable chance to actually provide substantial support. This depends mainly on the details of the resuscitation scenario and the causes of the OHCA. It is therefore important to explore if the volunteers are notified especially for resuscitation settings within the home situation and for help for OHCAs with a cardiac cause. This study aims to verify that the alert system is deployed in conditions for which it was initially developed by providing a description of the circumstances and causes of OHCAs, specifically where the citizen volunteers are involved.

## Methods

### Setting

We used data from a prospective registry consisting of all OHCAs in the Dutch province of Limburg (an area of approximately 2,153 km^2^ (831mi^2^) with 1.12 million inhabitants) during the period April 2012 to April 2014. Utstein recommendations and definitions were used [3–5]. The medical ethics committee of the Maastricht University Medical Centre approved the study (project number 114029).

### Resuscitation volunteer network in the study region

As outlined elsewhere [1], the basic professional procedure for an OHCA in the Netherlands consists of dispatching two ambulances to the scene, both manned by a paramedic and a basic life support (BLS)/automated external defibrillator (AED) trained driver, equipped with a defibrillator and requirements to provide advanced life support. Furthermore, the dispatch centralist can choose to activate the citizen volunteer system, a system where certified BLS/AED volunteers are notified by a text message. The dispatch centralist does not activate the system if the ambulance is already nearby or present at the scene, if the OHCA occurs in a (closed) public place with an on-site AED (such as shopping malls, sport venues etc.), if the OHCA is evidently of a non-cardiac aetiology or if the need for resuscitation is not recognised. The system uses the zip codes of the location of the victim and citizen rescuers to determine which volunteers are possibly closest to the victim, at least within a radius of 1 km (0.62 mile). In a 1:2 fashion, selected volunteers are notified to either go to the victim immediately or collect a system-registered AED first. To ensure a sufficient, but not excessive, number of volunteers, a maximum of 30 citizen rescuers are notified.

At the time of the study, 17 of the 24 dispatch centres in the Netherlands were using the system. In Limburg, both dispatch centres were active with a total of >9,000 volunteers (8.3/1,000 inhabitants).

### Data collection

We retrieved data from the following sources: 1. the dispatch centres from Limburg North and South, 2. their respective emergency medical services, 3. notified volunteers, 4. alert system organisation (*Hartslagnu*), 5. the six hospitals in Limburg, and 6. AED providers.

All notified volunteers received a questionnaire to obtain information about their attendance and, if applicable, about details of the resuscitation scenario. Medical history and post-resuscitation treatment were provided by the six hospitals in Limburg.

We assessed causes of OHCAs using information mostly from hospital records and discharge reports, autopsy reports, as well as from written information from the dispatch centre and ambulance personnel. All diagnoses were confirmed by one of the authors, a senior cardiologist (A.G.).

### Definitions

Acute coronary syndromes were cases with documented ST-elevation myocardial infarction or non-ST-elevation myocardial infarction. Cases with previous coronary revascularisation or old myocardial infarction were diagnosed as chronic coronary artery disease. Electrical heart diseases included tachycardia, mostly of ventricular origin; bradycardia, either unspecified or due to sinus bradycardia or atrioventricular block, or genetic forms such as Wolff-Parkinson-White, Brugada or long QT-syndrome. Structural heart disease consisted mostly of cases with hypertrophic or dilated cardiomyopathy. The diagnosis exsanguination included cases such as ruptured dissection/aneurysm or gastrointestinal bleed, and asphyxia was diagnosed in cases with respiratory insufficiency, pulmonary embolism or suffocation, mostly by choking.

### Statistical analysis

The distribution of causes of OHCA, patient characteristics and resuscitation settings were evaluated in the group of OHCAs in which the system was activated and compared with distribution in the group of OHCAs in which the system was not activated. Categorical variables were described as absolute numbers and percentages, and continuous variables as means with standard deviation or medians with interquartile range. The chi-square test was used to test for statistically significant differences in proportions between groups. For comparison of differences in continuous variables the t‑test for independent samples or the Mann-Whitney U test were used.

We used the statistical software package of SPSS (SPSS for Windows, version 22.0, SPSS Inc., Chicago, IL) to analyse the data.

## Results

**Fig. 1:**
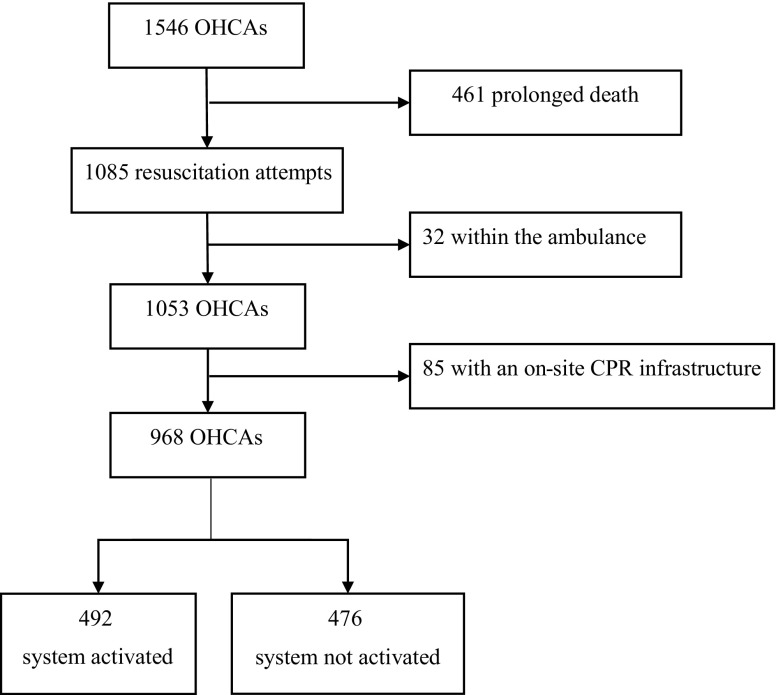
Flowchart of patient inclusion. *OHCA* out-of-hospital circulatory arrest, *CPR* Cardiopulmonary resuscitation

During the 24 months study period, 1,546 OHCAs were recorded. There were 461 victims with prolonged death and a resuscitation setting was present in 1,085 victims (including non-cardiac arrests and cases with a do-not-resuscitate policy). Arrests within the ambulance occurred in 32 instances. A total of 85 OHCAs occurred in closed public places with an on-site AED and local trained rescuers. Therefore, 968 cases were included for evaluation of causes of OHCA, patient characteristics and resuscitation settings in the OHCA population as encountered by the citizen rescuers. The system was activated in 492 arrests (50.8%) and not activated in 476 arrests (49.2%), as depicted in Fig. 1.

### Involvement of text message responders

Tab. 1 shows the baseline characteristics per scenario (activated versus not activated). The mean age of patients with OHCAs in which the system was activated was 67.9 (±14.1) and around 70% was male, similar to the distribution of age and sex in OHCAs in which the system was not activated.

**Table 1:** Population and resuscitation characteristics among the two different CPR scenarios

	Total *N* = 968	Activated *N* = 492	Not activated *N* = 476	*P*-value
*Demographic variables*				
Age, mean (SD), years, *n* = 966	67.1 (±15.4)	67.9 (±14.1)	66.2 (±16.6)	0.088
Gender, male, No. (%), *n* = 968	666 (68.8)	347 (70.5)	319 (67.0)	0.238
*Resuscitation variables*				
**Location of the arrest, No. (%), ** ***n*** **= 967**				<0.001
At home	727 (75.2)	413 (83.9)	314 (66.1)	
Outside home	240 (24.8)	79 (16.1)	161 (33.9)	
Witness, yes, No. (%), *n* = 968	748 (77.3)	369 (75.0)	379 (79.6)	0.086
**BLS started by, No. (%), ** ***n*** **= 959**				<0.001
Witness	297 (31.0)	178 (36.5)	119 (25.3)	
Bystander	193 (20.1)	119 (24.4)	74 (15.7)	
EMS	319 (33.3)	75 (15.4)	244 (51.8)	
TM responder	86 (9.0)	86 (17.6)	0 (0.0)	
First responder^a^	60 (6.3)	30 (6.1)	30 (6.4)	
None^b^	4 (0.4)	0 (0.0)	4 (0.8)	
*Ambulance times*				
**Time until arrival of first ambulance, ** ***n*** **= 953**				0.037
≤6 minutes	217 (22.8)	128 (26.6)	89 (18.9)	
7–8 minutes	234 (24.6)	116 (24.1)	118 (25.1)	
9–10 minutes	227 (23.8)	105 (21.8)	122 (25.9)	
≥11 minutes	275 (28.9)	133 (27.6)	142 (30.1)	
**Shock delivered, No. (%), ** ***n*** **= 968**	512 (52.9)	278 (56.5)	234 (49.2)	0.022

*BLS* basic life support, *CPR* cardiopulmonary resuscitation, *EMS* emergency medical system, *SD* standard deviation, *TM* text message

^a^On-duty police officer or firefighter notified to go to the resuscitation scene.

^b^Patients not being resuscitated because of a do-not-resuscitate policy

Regarding circumstances of OHCAs, citizen rescuers were more frequently involved in OHCAs within the home environment compared with resuscitations outside the home (83.9% vs 66.1%). A witness and/or bystander had already started resuscitation in 60.9% of cases (versus 41% in arrests where no volunteers were involved) and in approximately 18% the volunteers were the first to start BLS. The initial rhythm was shockable in 50% (versus 40.3% in arrests without involvement of volunteers) and in the former group also more frequently a shock was delivered.

Regarding the clinical setting of the OHCAs: in both study groups the majority of cases were found to have no cardiovascular history, thus the arrest being the first manifestation of cardiovascular disease.

In cases where the system was activated, the first ambulance arrived within 6 minutes in a mere 25% of cases. Delay between 6–11 minutes was recorded in approximately 50% and delay exceeding 11 minutes in approximately 25%. In the non-activated group the arrival times are unreliable because frequently the ambulance was already heading to the case before upscaling to the highest level of emergency due to the OHCA occurring during the ride.

Because the system was developed particularly for the treatment of arrests with a cardiac cause, we studied the distribution of causes among the two different scenarios. As expected, we found that citizen rescuers were more frequently involved in OHCAs with a cardiac cause and less frequently in cases with a non-cardiac cause. Cases were classified as unknown (251 cases in total), mostly when patients died before hospital arrival and no sufficient diagnostic information could be obtained.

Information on cardiac and non-cardiac causes is listed in Tab. 2. Basically, there were no differences in the distribution of causes between the activated and the non-activated group. The cause of the arrest was known in 345 and 372 cases in the activated and non-activated group, respectively. In 83.2% (287/345) of cases, volunteers were confronted with OHCAs with a cardiac cause, many being treatable. In a mere 16.8% (58/345), the OHCA was non-cardiac. These proportions were 67.5% (251/372) and 32.5% (121/372) without activation of the system.

**Table 2:** Distribution of specific causes^a^ among the two different CPR scenarios

	Total *N* = 717	Activated *N* = 345	Not activated *N* = 372	*P*-value
**Cardiac cause, No. (%)**	**538 (100)**	**287 (100)**	**251 (100)**	0.526
Acute coronary syndrome	187 (34.8)	96 (33.4)	91 (36.3)	
Chronic coronary artery disease	44 (8.2)	22 (7.7)	22 (8.8)	
Heart failure	62 (11.5)	37 (12.9)	25 (10.0)	
Electrical heart disease	42 (7.8)	18 (6.3)	24 (9.6)	
Structural heart disease	23 (4.3)	12 (4.2)	11 (4.4)	
VT/VF unspecified	180 (33.5)	102 (35.5)	78 (31.1)	
**Non-cardiac cause, No. (%)**	**179 (100)**	** 58 (100)**	**121 (100)**	0.405
Trauma	16 (8.9)	1 (1.7)	15 (12.4)	
Submersion	1 (0.6)	0 (0.0)	1 (0.8)	
Drug overdoses	4 (2.2)	1 (1.7)	3 (2.5)	
Asphyxia	78 (43.6)	26 (44.8)	52 (43.0)	
Exsanguination	21 (11.7)	8 (13.8)	13 (10.7)	
Suicide	7 (3.9)	3 (5.2)	4 (3.3)	
Other^b^	46 (25.7)	16 (27.6)	30 (24.8)	
PEA/asystole unspecified	6 (3.4)	3 (5.2)	3 (2.5)	

*CPR* cardiopulmonary resuscitation, *PEA* pulseless electrical activity, *VT* ventricular tachycardia, *VF* ventricular fibrillation

_a_In 251 cases the cause was unknown and therefore these cases are not included in this table

^b^Other includes cases such as cerebral causes or sepsis

Acute (33.4%) and chronic (7.7%) coronary artery disease were the most common cardiac causes. Heart failure was noted in 12.9%. In 35.5%, the initial rhythm was ventricular tachycardia (VT)/ventricular fibrillation (VF) unspecified, mostly patients who died at the scene and no further diagnostic information being available. Electrical and structural heart diseases were encountered by volunteers in 10.5% (30/287) of the cardiac cases versus 14% in the non-activated group.

In the 58 cases with a non-cardiac cause in which volunteers were involved, asphyxia (44.8%) was the most frequent cause and exsanguination was diagnosed in 13.8%. Trauma, drug overdoses and suicide were less likely to occur in the activated group and there was no resuscitation caused by submersion. Around 30% of the non-cardiac cases in the activated group had other underlying causes such as cerebral accidents or sepsis. In 3 cases in both groups the initial rhythm was pulseless electrical activity (PEA)/asystole, but the underlying causes could not be determined.

## Discussion

### Main findings

A population-based survey including all consecutive OHCAs showed that the majority of cases involving volunteers had a cardiac cause. In about 17% of cases with known aetiology, cardiopulmonary resuscitation (CPR) was needed after a collapse due to a non-cardiac cause. Treatable causes such as acute coronary syndrome was the most common cardiac cause. Around 60% of cases did not have a cardiovascular history, the arrest being the first manifestation of cardiac disease. This implies a good prognosis after successful resuscitation in the majority of patients, a feature already characterised in the early nineteen sixties as patients with ‘hearts too good to die’ [6].

### Study population and involvement of the text message volunteer

The system has been shown to increase survival in cardiac arrests if at least one volunteer responded [1]. In a minority of cases volunteers are notified for non-cardiac arrests, mostly due to asphyxia. In this situation, the involvement of volunteers could also be lifesaving by applying the Heimlich manoeuvre. Expectedly, volunteers are rarely confronted with trauma-related OHCA because centralists are instructed not to activate the system if the OHCA is obviously caused by trauma.

Zip code information about the resuscitation location is needed to activate the system, therefore OHCA occurring within the home environment was predominant (occurring in about 8 of 10 cases). Especially here support is needed not only because of the frequent occurrence of OHCA at home but also because of the more frequent absence of adequate CPR capabilities in that situation. Given its substantial contribution to survival, this system can be viewed as a new link in the chain of survival.

In about 60% of the cases a witness or bystander had already started BLS. Therefore, the system is helpful in supporting lay providers faced with an OHCA situation. In 18% of cases the volunteers were the first to start BLS. Although volunteers are BLS/AED certified, quick arrival of the emergency medical services is mandatory. In over 75% of cases, the ambulance arrival time exceeded 6 minutes, underscoring the importance of this system as a bridge to professional help. This is also supported by the higher percentage of shockable rhythms with involvement of the citizen rescuers, likely due to high quality BLS, helping to sustain VT/VF, rather than this to deteriorate in asystole [7].

In 42% of the OHCAs a volunteer alert would have been appropriate, but the alert system was not activated. The reasons why are currently being studied and are likely due to circumstances such as: the ambulance was already nearby or present at the scene, the OHCA occurred in an enclosed public place with an on-site AED, the OHCA was of a non-cardiac aetiology or the need for resuscitation was not recognised.

## Strengths and limitations

The strength of our study is that it concerns a population-based survey, performed in a well-defined geographical area, including all consecutive OHCA cases during a 2-year period. Although we tried to obtain accurate information from the notified volunteers by use of a questionnaire, it was practically impossible, due to the rapidly changing nature of a resuscitation setting, to retrieve exact numbers of responders and their arrival times at the location.

From the hospital records we could assess the medical history and the cause of the cardiac arrests of those being admitted to the hospital. This was not possible in 251 cases because these patients died at the scene. This limitation is of course inherent to a medical emergency occurring outside the hospital and with a low survival rate.

## Conclusion

The majority of OHCAs encountered by volunteers occur in the home environment, have a cardiac cause and involve ‘hearts too good to die’, underscoring the benefit of deploying citizen rescuers in programs to improve survival of OHCA.

## References

[1] Pijls RW, Nelemans PJ, Rahel BM, Gorgels AP (2016). A text message alert system for trained volunteers improves out-of-hospital cardiac arrest survival. Resuscitation.

[2] Pijls RW, Nelemans PJ, Rahel BM, Gorgels AP (2017). Factors modifying performance of a novel citizen text message alert system in improving survival of out-of-hospital cardiac arrest. Eur Heart J Acute Cardiovasc Care.

[3] Jacobs I, Nadkarni V, Bahr J (2004). Cardiac arrest and cardiopulmonary resuscitation outcome reports: update and simplification of the Utstein templates for resuscitation registries: a statement for healthcare professionals from a task force of the International Liaison Committee on Resuscitation (American Heart Association, European Resuscitation Council, Australian Resuscitation Council, New Zealand Resuscitation Council, Heart and Stroke Foundation of Canada, InterAmerican Heart Foundation, Resuscitation Councils of Southern Africa). Circulation.

[4] Peberdy MA, Cretikos M, Abella BS (2007). Recommended guidelines for monitoring, reporting, and conducting research on medical emergency team, outreach, and rapid response systems: an Utstein-style scientific statement: a scientific statement from the International Liaison Committee on Resuscitation (American Heart Association, Australian Resuscitation Council, European Resuscitation Council, Heart and Stroke Foundation of Canada, InterAmerican Heart Foundation, Resuscitation Council of Southern Africa, and the New Zealand Resuscitation Council); the American Heart Association Emergency Cardiovascular Care Committee; the Council on Cardiopulmonary, Perioperative, and Critical Care; and the Interdisciplinary Working Group on Quality of Care and Outcomes Research. Circulation.

[5] Perkins GD, Jacobs IG, Nadkarni VM (2015). Cardiac arrest and cardiopulmonary resuscitation outcome reports: update of the Utstein Resuscitation Registry templates for out-of-hospital cardiac arrest: a statement for healthcare professionals from a task force of the international liaison committee on resuscitation (American Heart Association, European Resuscitation Council, Australian and New Zealand Council on Resuscitation, Heart and Stroke Foundation of Canada, InterAmerican Heart Foundation, Resuscitation Council of Southern Africa, Resuscitation Council of Asia); and the American Heart Association Emergency Cardiovascular Care Committee and the Council on Cardiopulmonary, Critical Care, Perioperative and Resuscitation. Resuscitation.

[6] Beck CS, Leighninger DS (1960). Hearts too good to die—our problem. Ohio State Med J.

[7] Waalewijn RA, Nijpels MA, Tijssen JG, Koster RW (2002). Prevention of deterioration of ventricular fibrillation by basic life support during out-of-hospital cardiac arrest. Resuscitation.

