# Correction to: Circumstances and causes of sudden circulatory arrests in the Dutch province of Limburg and the involvement of citizen rescuers

**DOI:** 10.1007/s12471-018-1078-4

**Published:** 2018-01-30

**Authors:** R. W. M. Pijls, P. J. Nelemans, B. M. Rahel, A. P. M. Gorgels

**Affiliations:** 1 Department of Cardiology, CAPHRI school for Public Health and Primary Care, Maastricht University Medical Centre+, Maastricht, The Netherlands; 2 Department of Epidemiology, CAPHRI school for Public Health and Primary Care, Maastricht University Medical Centre+, Maastricht, The Netherlands; 3 Department of Cardiology, Viecuri Medical Centre for Northern Limburg, Venlo, The Netherlands

**Correction to:**

**Neth Heart J 2017**

10.1007/s12471-017-1057-1

In the version of the article originally published online, there was an error in the last section of the Discussion. It is stated that ‘In 42% of the OHCAs a volunteer alert would have been appropriate, but the alert system was not activated.’

The line should have read: ‘In 49% of the OHCAs a volunteer alert would have been appropriate, but the alert system was not activated.’

In the Results it is stated that ‘Basically, there were no differences in the distribution of causes between the activated and non-activated group’.

This sentence should have read: ‘Within the subgroups with cardiac cause versus non-cardiac cause, there were no differences in the distribution of causes between the activated and non-activated group.’

